# Indicators related to the rational use of medicines and its associated factors

**DOI:** 10.11606/S1518-8787.2017051007137

**Published:** 2017-09-22

**Authors:** Marina Guimarães Lima, Juliana Álvares, Augusto Afonso Guerra, Ediná Alves Costa, Ione Aquemi Guibu, Orlando Mario Soeiro, Silvana Nair Leite, Margô Gomes de Oliveira Karnikowski, Karen Sarmento Costa, Francisco de Assis Acurcio

**Affiliations:** I Departamento de Farmácia Social. Faculdade de Farmácia. Universidade Federal de Minas Gerais. Belo Horizonte, MG, Brasil; II Instituto de Saúde Coletiva. Universidade Federal da Bahia. Salvador, BA, Brasil; III Departamento de Saúde Coletiva. Faculdade de Ciências Médicas. Santa Casa de São Paulo. São Paulo, SP, Brasil; IV Faculdade de Ciências Farmacêuticas. Pontifícia Universidade Católica de Campinas. Campinas, SP, Brasil; V Departamento de Ciências Farmacêuticas. Universidade Federal de Santa Catarina. Florianópolis, SC, Brasil; VI Faculdade de Ceilândia. Universidade de Brasília. Brasília, DF, Brasil; VII Núcleo de Estudos de Políticas Públicas. Universidade Estadual de Campinas. Campinas, SP, Brasil; VIII Programa de Pós-Graduação em Saúde Coletiva. Departamento de Saúde Coletiva. Faculdade de Ciências Médicas. Universidade Estadual de Campinas. Campinas, SP, Brasil; IX Programa de Pós-Graduação em Epidemiologia. Faculdade de Medicina. Universidade Federal do Rio Grande do Sul. Porto Alegre, RS, Brasil

**Keywords:** Drug Utilization, statistics & numerical data, Quality Indicators, Health Care, Management Indicators, Pharmaceutical Services, Primary Health Care, Health Services Research, Unified Health System, Uso de Medicamentos, estatística & dados numéricos, Indicadores de Qualidade em Assistência à Saúde, Indicadores de Gestão, Assistência Farmacêutica, Atenção Primária à Saúde, Pesquisa sobre Serviços de Saúde, Sistema Único de Saúde

## Abstract

**OBJECTIVE:**

To evaluate indicators related to the rational use of medicines and its associated factors in Basic Health Units.

**METHOD:**

This is a cross-sectional study carried out in a representative sample of Brazilian cities included in the *Pesquisa Nacional sobre Acesso, Utilização e Promoção do Uso Racional de Medicamentos – Serviços, 2015* (PNAUM – National Survey on Access, Use and Promotion of Rational Use of Medicines – Services, 2015). The data were collected by interviews with users, medicine dispensing professionals, and prescribers; and described by prescription, dispensing, and health services indicators. We analyzed the association between human resources characteristics of pharmaceutical services and dispensing indicators.

**RESULTS:**

At national level, the average number of medicines prescribed was 2.4. Among the users, 5.8% had antibiotic prescription, 74.8% received guidance on how to use the medicines at the pharmacy and, for 45.1% of users, all prescribed medicines were from the national list of essential medicines. All the indicators presented statistically significant differences between the regions of Brazil. The dispensing professionals that reported the presence of a pharmacist in the unit with a working load of 40 hours or more per week presented 1.82 more chance of transmitting information on the way of using the medicines in the dispensing process.

**CONCLUSION:**

The analysis of prescription, dispensing, and health services indicators in the basic health units showed an unsatisfactory proportion of essential medicines prescription and limitations in the correct identification of the medicine, orientation to the patients on medicines, and availability of therapeutic protocols in the health services.

## RESUMO

**OBJETIVO:**

Avaliar indicadores relacionados ao uso racional de medicamentos e seus fatores associados em unidades básicas de saúde.

**MÉTODOS:**

Estudo transversal realizado em amostra representativa de municípios do Brasil incluídos na Pesquisa Nacional sobre Acesso, Utilização e Promoção do Uso Racional de Medicamentos – Serviços, 2015. Os dados foram coletados por meio de entrevista com usuários, profissionais dispensadores de medicamentos e prescritores; e descritos por meio de indicadores de prescrição, dispensação e de serviços de saúde. Realizou-se análise da associação entre características dos recursos humanos dos serviços farmacêuticos e indicadores de dispensação.

**RESULTADOS:**

Em nível nacional, o número médio de medicamentos prescritos foi de 2,4. A proporção de usuários com prescrição de antibiótico foi de 5,8%, 74,8% dos usuários receberam orientações sobre medicamentos na farmácia e para 45,1% usuários todos os medicamentos prescritos eram da relação nacional de medicamentos essenciais. Todos os indicadores apresentaram variações estatisticamente significantes entre as regiões do Brasil. Os dispensadores que relataram a presença de farmacêutico na unidade com carga horária igual ou superior a 40 horas semanais apresentaram 1,82 mais chance de transmitir orientações sobre o modo de usar dos medicamentos no processo de dispensação.

**CONCLUSÕES:**

A análise de indicadores de prescrição, dispensação e de serviços de saúde nas unidades básicas de saúde mostrou proporção insatisfatória de prescrição de medicamentos essenciais e limitações na identificação correta do medicamento, orientação aos pacientes sobre medicamentos e de disponibilidade de protocolos terapêuticos nos serviços de saúde.

## INTRODUCTION

Rational use of medicines requires users to use the appropriate medicine for their clinical condition in doses that meet their individual health needs, during an appropriate period and at the lowest cost to themselves and the community^20^.

The non-rational use of medicines may have a negative impact on population health, including avoidable adverse events and microbial resistance^24^. Adverse medicine events are estimated to account for 3.5% of hospital admissions^6^. According to one study, the occurrence of these events resulted in health services expenditure of about $ 21 million per 100,000 adult population^11^_._

The evaluation of the activities of Pharmaceutical Services (PS) is fundamental to promote the access and rational use of medicines. To assist in the PS evaluation, the World Health Organization (WHO) has developed indicators that can be used reproducibly so that methods are reliable and comparable across different studies and locations^15^. According to a document published in 2007, PS monitoring and evaluation can be performed at three levels. Level I concerns aspects of structure and organization process of the pharmaceutical industry. Level II targets the results of the national drug policy and is measured in public and private services and in households, in addition to the domains of access, quality, and rational use of medicines. The evaluation is conducted by a survey based on visits to state and municipal pharmacy supply centers; to public health units that perform ambulatory care and dispensing of medicines; and to private pharmacies of retail trade, being adaptable to the type of study that will be conducted. Level III details specific aspects of the organization of the pharmaceutical sector^23^_._

According to a systematic review involving 900 studies conducted in 104 countries, the analysis of indicators on the rational use of medicines indicated that the inappropriate use of pharmaceuticals remains a public health problem^12^. This review included studies performed in public primary health care services. A similar scenario was observed in a multicenter study conducted in Brazil in 2004, which observed 40.1% of antibiotic prescriptions, 6.9% of injectable medicines, and 78.3% of medicines present in the list of essential medicines^9^ ^,^ ^16^. To evaluate the rational use of medicines in Brazil, current data are needed, from a representative sample of the Brazilian population that uses *Unidades Básicas de Saúde* (UBS – Basic Health Units).

The *Pesquisa Nacional sobre Acesso, Utilização e Promoção do Uso Racional de Medicamentos – Serviços* (PNAUM – National Survey on Access, Use and Promotion of Rational Use of Medicines – Services) aimed to characterize the organization of pharmaceutical services in the primary care of the Brazilian Unified Health System (SUS) – to promote the access and rational use of medicines –, as well as to identify and discuss the factors that interfere in the consolidation of pharmaceutical services in the cities.

This study integrates PNAUM – Services and aims to evaluate indicators related to the rational use of medicines in the UBS and its associated factors.

## METHODS

This study is part of PNAUM – Services, a cross-sectional, exploratory, evaluative study, composed of a survey of information in a representative sample of cities, primary health care services, users, physicians, and professionals responsible for dispensing of medicines in the five regions of Brazil. The sampling plan considered the several study populations and estimated the various sample sizes for each of these populations^1^. The sample size was estimated by algebraic expression^a^. The sample sizes adopted in each region were 120 municipalities, 300 health services, and 1,800 users. The sample was stratified in capitals (26 and the Federal District); biggest cities (0.5% biggest cities in the region, totaling 27) and smallest cities (546 cities chosen by lot). From these 120 selected municipalities, 60 were selected by region, totaling 300 in the country, in which the health services were chosen by lot. Health posts, health centers or UBS, and mixed units were included in the lot, according to the *Cadastro Nacional de Estabelecimentos de Saúde* (CNES – National Register of Health Establishments).

Face-to-face interviews were conducted with users, physicians, and those responsible for dispensing of medicines in the primary health care services, as well as telephone interviews with those responsible for pharmaceutical services in the cities, using a structured questionnaire specific to each category. The observation of the facilities of pharmaceutical services and availability of medicines were verified by observation script. A manual and a glossary of technical terms were developed for each research instrument. After the training of the interviewers, a pretest was carried out, involving cities with different population sizes, aiming to validate and improve the instruments. The data were collected from July 2014 to May 2015.

PNAUM considered as dispensers the professionals responsible for delivering the medicines to users, who may be pharmacists, nurses, nursing assistants, pharmacy assistants, or other professional category.

The data were described by prescription, dispensing, and health services indicators, outlined for the study. The list of indicators and the criteria for their calculation were based on those recommended by the WHO to evaluate the rational use of medicines^23^, with adaptations and propositions carried out by the researchers (Table 1). The option of using indicators based on those recommended by WHO was performed to allow the comparison of PNAUM results with those obtained by national and international studies.

**Table 1:** List of prescription, dispensing, and health services indicators related to the rational use of medicines. National Survey on Access, Use and Promotion of Rational Use of Medicines – Services, 2015.

Indicator	Criteria for Calculation
Prescription	
Average number of prescription medicines	We considered the number of medicines used by users who had at least one medicine prescribed by a doctor or dentist for the calculation of the average.
Proportion of users with antibiotic prescription	We considered the number of users who used at least one antibiotic as numerator and the number of users who used at least one medicine prescribed by a doctor or dentist as denominator. We considered as antibiotics the bacteriostatic and bactericidal antibacterial medicines, in systemic and topical use presentations. We considered the following categories in the Anatomical-Therapeutic-Chemical classification level (ATC): D06A-Antibiotics for topical use, D06BA-Sulfonamides, D06C-Antibiotics and chemotherapeutics combinations, J01-Antibacterials for systemic use, and J04-Antimycobacterials.
Proportion of users with injectable prescription	The numerator was the number of users who used at least one injectable medicine and the denominator was the number of users who used at least one medicine prescribed by a doctor or dentist.
Proportion of users with all prescribed medicines present in the national list of essential medicines	The numerator was the number of users with all prescribed medicines present in the National List of Essential Medicines of 2013, in force during the period of data collection. The denominator was the number of medicines prescribed by a doctor or dentist.
Dispensing	
Percentage of professionals dispensing medicines identified with name and dose	We considered the number of professionals who reported dispensing medicines identified with name and dose as numerator and the number of professionals that act in dispensing medicines and who answered the questionnaire item on this topic as denominator.
Proportion of users who received guidance on medicines at the pharmacy	We considered the number of users who reported receiving guidelines on medicines at the pharmacy as numerator and the number of users who had at least one medicine prescribed by a doctor or dentist as denominator.
Health services	
Availability of relevant therapeutic protocols in the medical offices, reported by physicians	We considered the number of doctors who reported the presence of relevant therapeutic protocols in the health units as numerator and the number of doctors interviewed as denominator.
Availability of a copy of the local or national list of essential medicines, reported by the dispenser	We considered the number of dispensing professionals who reported the availability of a copy of the local or national list of essential medicines in the unit as numerator and the number of dispensing professionals interviewed as denominator.

Source: PNAUM – Services, 2015.

A descriptive analysis of the variables used in the study was performed. The indicators were described according to regions of Brazil. The comparison of the indicators between the regions was done with Chi-square test for the categorical variables and with ANOVA for the continuous ones. We analyzed the association between human resources characteristics of pharmaceutical services and indicators of dispensing related to the rational use of medicines by logistic regression, with calculation of the odds ratio (OR). We considered a 5% statistical significance level and 95.0% confidence interval. SPSS version 22.0 was used for statistical analyses.

Participants signed an informed consent form. PNAUM – Services was approved by the National Research Ethics Committee of the National Health Council, by Opinion no. 398.131/2013.

## RESULTS

PNAUM – Services interviewed 8,803 users, 1,585 doctors, and 1,139 professionals responsible for the dispensing of medicines in the UBS of the five regions of Brazil. Among the users, 6,010 (68.3%) used at least one medicine prescribed by a physician or dentist and 4,890 (55.5%) had at least one chronic disease. The proportion of medicines present in the *Relação Nacional de Medicamentos Essenciais* (Rename – National List of Essential Medicines) was 55.2%. Table 2 shows the values of prescription, dispensing, and health services indicators related to the rational use of medicines. All indicators presented statistically significant differences between the regions. The average number of medicines prescribed in Brazil (2.4) was higher than the average value in the North (1.8), Midwest (2.0), and Northeast (2.2), but lower than in the South (2.9). The lowest proportion of users with antibiotic prescription was in the Southeast (3.8%) and the highest in the North (10.1%). The percentage of users who received guidance on medicines ranged from 71.4% in the South to 85.3% in the Midwest. The availability of a copy of Rename was reported by 80.5% of dispensing professionals in the North region and by 95.1% in the Southeast.

**Table 2:** Prescription, dispensing, and health services indicators related to the rational use of medicines according to the Region of Brazil. National Survey on Access, Use and Promotion of Rational Use of Medicines – Services, 2015.

Indicator	Midwest	Northeast	North	Southeast	South	Brazil	p
Prescription							
Average number of medicines prescribed^a^	2.0	2.2	1.8	2.2	2.9	2.4	< 0,01
Proportion of users with antibiotic prescription^a^ (%)	6.6	5.4	10.1	3.8	6.9	5.8	< 0,01
Proportion of users with injectable prescription^a^ (%)	5.8	4.8	8.1	6.0	6.5	6.0	< 0,01
Proportion of users with all prescribed medicines present in the national list of essential medicines^a^ (%)	47.0	45.6	46.5	52.2	38.7	45.1	< 0,01
Dispensing^b^							
Percentage of professionals dispensing medicines identified by name and dose (%)^b^	65.7	64.8	65.4	72.5	69.4	67.4	< 0,01
Proportion of users who received guidance on medicines at the pharmacy^a^ (%)	85.3	80.5	80.9	72.9	71.4	74.8	< 0,01
Health services							
Availability of relevant therapeutic protocols in medical offices, reported by physicians (%)	60.2	57.3	51.1	49.5	53.6	46.2	< 0,01
Availability of a copy of the local or national list of essential medicines, reported by the dispenser (%)	87.0	87.5	80.5	95.1	91.3	89.5	< 0,01

^a^ Indicators calculated considering the number of patients who had at least one prescribed medicine as denominator (N = 6010)

^b^ Calculated based on the professionals who answered the question

Source: PNAUM – Services, 2015.

The proportion of professionals who reported always transmitting information on how to use the medicines at the time of delivery of the pharmaceutical product was 90.9%. The dispensing professionals who participated in PS training in the two years prior to the interview had 1.49 more chance of dispensing medicines identified with name and dose (OR = 1.49, 95%CI, 1.36-1.62, p=0.00) and were less likely to provide guidance on how to use the products (OR = 0.86, 95%CI 0.77-0.96, p=0.01). The dispensing professionals whose units have full-time pharmacist were 1.82 times more likely to provide guidelines on the use of medicines (OR = 1.82, 95%CI 1.11-2.99, p=0.02). We observed no statistically significant association between dispenser performance in units with full-time pharmacist and dispensing of medicines identified with name and dose (OR = 1.17, 95%CI 0.67-2.03, p=0.58).

## DISCUSSION

The analysis of prescription, dispensing, and health services indicators in the UBS pointed to aspects that should be considered in the consolidation of the *Política Nacional de Assistência Farmacêutica* (PNAF – National Policy of Pharmaceutical Services) to promote the rational use of medicines in primary health care.

The mean number of prescribed medicines observed in this study (2.4) was similar to that found by a multicenter study conducted in Brazil in 2004^9^ (2.3) and was higher than the range of values considered standard for the indicator (less than 2)^7^ ^,^ ^22^. A systematic review of studies conducted in Africa identified an average prescription of medicines of 3.1^14^. In Brazil, this indicator presented significant regional variations, with 2.2 in the Northeast and 2.9 in the South, a situation that may be related to socioeconomic differences between regions. This result is in line with another cross-sectional study conducted in a sample of adult and older adult users of UBS, in which the prevalence of access to medicines for continuous use was higher in the South region than in the Northeast^17^. The authors attributed the difference to a higher proportion of users belonging to higher socioeconomic levels in the South^17^.

The analysis of the frequency of antibiotic prescriptions is performed to assess their overuse^23^, which leads to microbial resistance in the population^13^. In this study, the proportion of patients with antibiotic prescriptions was 5.8%, lower than the average values of 37% for Latin America^12^ and 46.8% for Africa^13^ observed in systematic reviews^12^ ^,^ ^14^. The value of this indicator was also lower than the established standard (less than 30%)^7^ ^,^ ^22^, suggesting that the proportion of antibiotic prescriptions is satisfactory in the UBS user population. In 2011, control of the dispensing of antimicrobial medicines was encouraged by the publication of RDC no. 20/2011 of the National Sanitary Surveillance Agency, which now requires retention of prescription in establishments dispensing products of this therapeutic class. Although the legislation regulates especially the dispensing process, we can assume that it has influenced the behavior of prescribers to increase caution regarding the prescription of antibiotics. However, this hypothesis and other possible factors associated with the use of antibiotics should be evaluated in a study outlined for this purpose. We observed regional variations in the proportion of antibiotic prescriptions, which was highest in the North region. This situation may have occurred because of the epidemiological profile of the region, with lower prevalence of chronic diseases compared to others with more favorable socioeconomic conditions, such as South and Southeast^3^.

A systematic review of international studies showed a frequency of injectable medicines prescriptions of about 20%^12^. In this study, injectable medicines were prescribed to 6.0% of users, a value similar to the proportion observed by a Brazilian study carried out in three different states^9^. Because of the risk of complications for incorrect administration of parenteral medicines, the prescription of injectable products has been restricted to procedures performed in the UBS itself and to medicines that are not available in the oral form in pharmaceutical market, such as insulin.

The use of standardized lists of medicines in health systems contributes to the promotion of quality of care when products are selected according to criteria of health need, efficacy, safety, quality, and cost^22^. In this study, the proportion of medicines present in Rename was 55.2%, lower than that observed in Latin America (71.4%)^12^, Africa (88.0%)^14^, and in another Brazilian study^9^ (78.3%) carried out in 2004. The standard considered ideal for this indicator is 100%^7^, so that the proportion found by PNAUM – Services was unsatisfactory.

We observed that, for 45.1% of users, all prescribed medicines were included in Rename. According to a study carried out in a region of China, the doctor’s knowledge about medicines was associated with a higher prescription of essential medicines^19^. Researches on factors associated with the prescription of essential medicines in the Brazilian context are needed to subsidize policies of permanent education for prescribing professionals in SUS. Despite the importance of adopting the relation of essential medicines for the rational prescription of medicines, the limitations of Rename should be emphasized. A Brazilian research related Rename’s medicines to studies of the Global Burden of Disease in Brazil^10^. According to an analysis done for Rename’s 2012 edition, some causes of disability-adjusted life years (DALY) have not been fully addressed by the medicines on the list, such as oral conditions, cancer, and psychiatric diseases^10^.

The identification of medicines with name and dose at the time of dispensing was reported by 67.4% of dispensing professionals. A Brazilian study pointed out that 95.2% of the medicines offered by SUS had data related to name, concentration, manufacturer, batch, and expiration period^9^. A study carried out in the primary health care of Botswana found that 74% of medicines dispensed at health posts were identified by name^4^. To ensure that the required amount of tablets is provided for the treatment of patients in the UBS, the blisters are cut, which leads to problems in medicine identification. The absence of identification of the pharmaceutical products in the primary packaging observed in this study can lead to medication errors, such as the use of expired medicines or exchange for another product by the user^5^.

The dispensing of medicines involves patient orientations that contribute to the rational use of medicines, such as how to use them, time of treatment, major adverse reactions, and interactions with medicines and food^13^. The transmission of medicine guidelines is fundamental for the adherence to treatment and success of pharmacological therapy^21^. In our study, 74.8% of users reported having received information at the pharmacy on how to use the medicines, a proportion lower than that of a study conducted in a Brazilian city (92.5%)^18^. The professionals who reported the presence of a full-time pharmacist in the UBS had a greater chance of transmitting information to users. Dispensing professionals at Brazilian UBS may be pharmacists or assistants supervised by pharmacists or nurses. The presence and performance of the pharmacist in a weekly workload of 40 hours helps this professional so that he can guide or train assistants for the guidance on medicines.

The comparison of our results with others is difficult because of the differences in the adopted methodology: PNAUM questioned whether dispensers always delivered the medicines identified with name and dose to the patients. Dispensers who had PS training were more likely to dispense the medicines identified by name and dose, showing the importance of continuous health education actions for professionals involved in dispensing pharmaceuticals. In a contradictory way, professionals who participated in training were less likely to give guidance on how to use the medicines. This contradiction suggests that the training may have mainly addressed administrative procedures of dispensing as a matter of priority, but insufficiently the clinical aspects, such as patient orientation. However, this hypothesis must be tested by studies evaluating educational activities on the rational use of medicines regarding content, employed methods, and impact.

The availability of a Rename was of 89.5%, similar to that found by a research carried out in health facilities in Saudi Arabia (90%)^8^ and lower than that observed by a study done in Pakistan (100%)^2^. However, the methodology used to estimate this indicator was different between the studies.

The therapeutic protocols are based on scientific evidence and reflect a consensus on the treatment of first choice for several clinical conditions, contributing to the promotion of rational prescription. In this study, 46.2% of physicians reported the availability of therapeutic protocols in the medical offices. This indicator was not evaluated in recent studies of the literature, but a Brazilian study conducted in 2004 indicated the availability of protocols for the treatment of tuberculosis in 43.3% of health units^9^.

Among the limitations of the study, we highlight that the data on the accomplishment of medicines identification and orientation to the users in the dispensing process were collected by the professionals’ report and not by direct observation. The methodology used to estimate the indicators in PNAUM – Services differed in some situations from that used in other studies, which made it difficult to compare the results. Despite the limitations, our study presented an unprecedented panorama in the literature on the rational use of medicines in a representative sample of the UBS user population in Brazil.

Thus, we observed an unsatisfactory proportion of prescription of essential medicines and limitations in the correct identification of the medicine, guidance to patients on medicines, and availability of therapeutic protocols in the health services. The statistically significant difference in the values of the indicators between the regions of Brazil suggests that regional specificities should be considered in the formulation of policies aimed at increasing the rationality of the use of pharmaceuticals. The unsatisfactory proportion of prescription of essential medicines in the UBS points out the need for training SUS prescribers on the rational use of medicines. Regarding the dispensing process, educational activities for professionals of primary health care units and their supervision by full-time pharmacists may help users to use the right medicine for their clinical condition and have access to guidelines on their pharmacological treatment. Measures that qualify health, prescription, and dispensing services are needed to promote the rational use of medicines, which is one of the main goals of PNAF.

## Footnotes

a The sample was estimated by the algebraic expression $n_{0}\frac{P\left(1-p\right)}{{\left(d/z\right)}^{2}}\cdot deff$ , where: P = 0.50 is the proportion of individuals to be estimated for being the one that leads to the largest sample size; Z = 1.96 is the value in the reduced normal curve for the 95% confidence intervals; deff is the effect of the design; d is the sampling error in percentage points.
